# The interaction between iodinated X‐ray contrast agents and macrocyclic GBCAs provides a signal enhancement in T_1_‐weighted MR images: Insights into the renal excretion pathways of Gd‐HPDO3A and iodixanol in healthy mice

**DOI:** 10.1002/mrm.29190

**Published:** 2022-03-07

**Authors:** Enza Di Gregorio, Francesca Arena, Eliana Gianolio, Giuseppe Ferrauto, Silvio Aime

**Affiliations:** ^1^ Molecular Imaging Center, Department of Molecular Biotechnology and Health Sciences University of Turin Turin Italy; ^2^ Istituto di ricovero e cura a carattere scientifico Naples Italy

**Keywords:** contrast agents, Gd‐complexes, iodinated X‐ray, magnetic resonance imaging, X‐ray CT

## Abstract

**Purpose:**

This work aims to investigate the supramolecular binding interactions that occur between iodinated X‐ray contrast agents (CAs) and macrocyclic gadolinium (Gd)–based MRI contrast agents (GBCAs). This study provides some new insights in the renal excretion pathways of the two types of imaging probes.

**Methods:**

The water‐proton relaxivities (*r*_1_) of clinically approved macrocyclic and linear GBCAs have been measured in the presence of different iodinated X‐ray contrast agents at different magnetic field strengths in buffer and in serum. The in vivo MRI and X‐ray CT of mice injected with either Gd‐HPDO3A or a Gd‐HPDO3A + iodixanol mixture were then acquired to assess the biodistribution of the two probes.

**Results:**

A significant increase in *r*_1_ (up to approximately 200%) was observed for macrocyclic GBCAs when measured in the presence of an excess of iodinated X‐ray CAs (1:100 mol:mol) in serum. The co‐administration of Gd‐HPDO3A and iodixanol in vivo resulted in a marked increase in the signal intensity of the kidney regions in T_1_‐weighted MR images. Moreover, the co‐presence of the two agents resulted in the extended persistence of the MRI signal enhancement, suggesting that the Gd‐HPDO3A/iodixanol adduct was eliminated more slowly than the typical washing out of Gd‐HPDO3A.

**Conclusions:**

The reported results show that it is possible to detect the co‐presence of iodinated agents and macrocyclic GBCAs in contrast‐enhanced MR images. The new information may be useful in the design of novel experiments toward improved diagnostic outcomes.

## 1 INTRODUCTION

Contrast agents (CAs) play a central role in the diagnostic applications of CT and MRI.^1^, ^2^, ^3^ In CT, the most frequently used CAs are currently chemicals that are based on the tri‐iodo‐benzene moiety and functionalized with hydrophilic groups to endow them with the required high aqueous solubility.^4^, ^5^, ^6^, ^7^ Magnetic resonance imaging CAs are paramagnetic complexes of the Gd^3+^ ion.^8^ Because Gd^3+^ is toxic, gadolinium (Gd)–based contrast agents (GBCAs) consist of complexes with octadentate ligands from the polyaminocarboxylate class.^9^, ^10^, ^11^ They are commonly classified into two groups: the group containing linear ligands, whose reference system is diethylenetriamine penta‐acetic acid (DTPA), and the group containing macrocyclic ligands, such as tetraazacyclododecanetetraacetic acid (DOTA) and HPDO3A.^12^, ^13^ In general, it is assumed that the distribution of CT and MRI CAs that are injected intravenously is analogous due to their common hydrophilicity, although their pharmacokinetics may differ slightly.^14^ Herein, we report that it is possible to report on the co‐presence of iodinated agents and macrocyclic GBCAs in contrast‐enhanced MR images. In fact, the co‐presence of the two types of CA resulted in an enhancement in signal intensity in the corresponding T_1_‐weighted MR images and in density contrast in the CT images of the kidneys.

The rationale for this behavior can be found in the recently reported observations^15^ of the occurrence of hydrophobic interactions between the tetra‐aza‐macrocyclic moiety of macrocyclic GBCAs and pyrene derivatives, which contain sulfonated and OH moieties on their external perimeter. We report that an analogous hydrophobic interaction can be generated when the pyrene derivatives are replaced by the tri‐iodo‐benzene moiety.

## 2 METHODS

### 2.1 Chemicals

**FIGURE 1:**
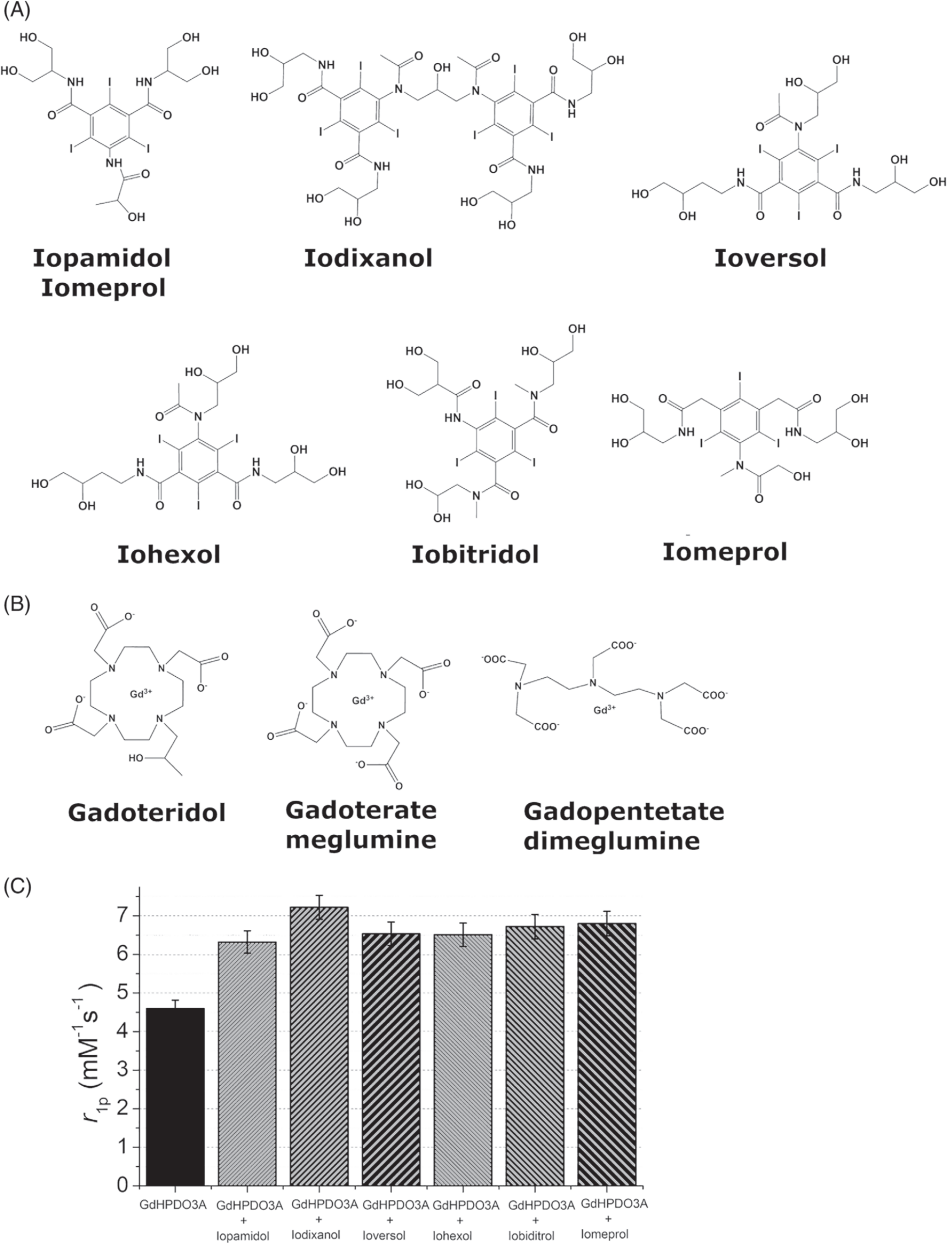
Chemical structures of the iodinated X‐ray contrast agents (A) and of the gadolinium (Gd)–based MRI contrast agents (B). (C) Relaxivity plot of Gd‐HPDO3A (1 mM) in the presence of the iodinated X‐ray contrast agents (100 mM) (B_0_ = 0.5 T, pH = 7.1 ± 0.1, T = 25°C)

The following clinically approved iodinated CT contrast agents were used in this study: (1) iopamidol (Isovue; Bracco Imaging), (2) iodixanol (Visipaque; GE Healthcare), (3) ioversol (Optiray 300; Guerbet), (4) iohexol (Omnipaque; GE Healthcare), (5) iobitridol (Xenetix; Guerbet), and iomeprol (Iomeron; Bracco Imaging) (chemical structures shown in Figure 1A).

The following clinically approved Gd‐based CAs were used in this study: (1) gadoteridol (ProHance; Bracco Imaging, Gd‐HPDO3A), (2) gadoterate meglumine (Dotarem; Guerbet, Gd‐DOTA), and (3) gadopentetate dimeglumine (Magnevist; Bayer, Gd‐DTPA) (chemical structures shown in Figure 1B).

Sodium chloride, HEPES, and all other chemicals were purchased from Sigma Aldrich–Merck (Darmstadt, Germany) and used without further purification. Human serum (Seronorm) was purchased from Sera (Norway).

### 2.2 Relaxometric measurements

The longitudinal proton relaxation rates (*R*_1_ = 1/T_1_) of aqueous solutions of the Gd‐complexes (1 mM; Gd‐HPDO3A, Gd‐DOTA, and Gd‐DTPA) were determined in the presence of various iodinated X‐ray CAs (0–300‐mM range). The relaxometric measurements were carried out at 298 K or 310 K in a HEPES/NaCl buffer solution (HEPES, 3.8 mM; NaCl, 150 mM) at pH 7.1 ± 0.1 on a Stelar SpinMaster Relaxometer (Mede, PV, Italy) operating at 21.5 MHz, using the inversion‐recovery method (16 experiments, two scans). The reproducibility of the T_1_ data was ±0.5%.

To mimic in vivo conditions, the relaxivity values were also acquired in a serum solution (Seronorm; SERO, Billingstad, Norway).

The effect of microviscosity was assessed by acquiring relaxometric measurements of Gd‐HPDO3A and Gd‐DTPA in the presence of glycerol (40%, vol/vol).

### 2.3 Animal handling

For the in vivo imaging experiments, 10‐week‐old Balb/c male mice (Charles River Laboratories, Calco, Bergamo, Italy) were used. The mice were bred at the animal house at the Center of Excellence for Preclinical Imaging at the University of Turin. They were kept in standard housing conditions with standard rodent chow, water available ad libitum, and a 12‐hour light/dark cycle.

All procedures involving animals were performed in accordance with national and international laws on the use of experimental animals (L.D. 26/2014; Directives 2010/63/EU) under Ministerial Authorization (Project Research No. 229/2016‐PR).

### 2.4 Magnetic resonance imaging

Mice were used upon anesthetization with sevoflurane gas 0.5% (O_2_ 95%) and placed supine in a solenoid Tx/Rx coil with an inner diameter of 3.5 cm. Breathing rate was monitored throughout the in vivo MRI experiments using a respiratory gate–containing probe (SAII Instruments, Stony Brook, NY, USA).

Mice were either injected with 0.05 mmol/Kg of Gd‐HPDO3A or a mixture of Gd‐HPDO3A and iodixanol (0.05 mmol/Kg Gd‐HPDO3A and 5 mmol/Kg iodixanol).

The volume of the intravenously injected bolus was 0.350 ml. It consisted of 421 mM iodixanol (4 gI/kg) + 4.2 mM PhoHance (0.05 mmol/kg).

The MR images were acquired on a 1T MR scanner (Icon, Bruker) at room temperature. After the acquisition of the scout image, T_1_‐weighted images were obtained using a rapid acquisition with refocused echoes sequence with the following parameters: TR = 380 ms, TE = 14.6 ms, flip angle = 180°, number of averages = 4, slice thickness = 1 mm, number of slices = 10, matrix size = 256 × 256, FOV = 3.5 cm, in‐plane resolution = 0.137 mm/pixel, and total acquisition time = 2 min and 25 s.

Magnetic resonance image acquisitions were performed *before* and 20, 60, and 180 min *after* the injection of either ProHance or the iodixanol‐ProHance mixture.

The MRI signal intensity was analyzed in manually drawn regions of interest (ROIs) in different kidney regions (cortex, medulla, and ureters/renal pelvis; ROI shown in Supporting Information Figure S1). A glass tube containing a standard solution of Gd‐HPDO3A (0.5 mM) was placed in the FOV in close proximity to the mouse body as an internal reference.

### 2.5 Computed tomography images

For CT imaging experiments, animals were anesthetized with sevoflurane gas 0.5% (O_2_ 95%) and maintained warmed at 37°C during the acquisitions. A total volume of 0.4 ml of 4.0 g/kg iodixanol or iodixanol/Gd‐HPDO3A mixture (4.0 g/kg/0.05 mmol/kg) was manually injected, in about 25 s, in the tail vein of mice using a 27‐gauge intravenous catheter. The CT imaging was performed *before* and 10, 40, and 180 min *after* injection of the contrast media.

The CT images were acquired on a GMI‐Triumph trimodality preclinical scanner operating at 75 Kv, with 512 views and ×2.3 magnification. Attenuation is reported as Hounsfield units (HU), which were calculated as follows:

$$\mathrm{HU}=1000\times \frac{\mu -\mu_{\text{water}}}{\mu_{\text{water}}-\mu_{\mathrm{air}}}$$

where *μ*_water_ and *μ*_air_ are the linear attenuation coefficients of water and air, respectively.

An example of ROIs drawn in cortex, medulla, and ureters/renal pelvis is reported in Supporting Information Figure S1.

## 3 RESULTS

Nonionic iodinated molecules are used widely in X‐ray and CT investigations (chemical structure shown in Figure 1A). They consist of a benzoic acid derivative containing three iodine atoms per molecule, and of several evenly distributed hydroxyl groups.

The latter contribute to reducing osmolality, to improving hydrophilicity and to restricting access to the lipophilic areas of the molecule. Nonionic dimers (e.g., iodixanol) contain a double‐benzene ring with six iodine atoms.^16^, ^17^, ^18^, ^19^, ^20^

Our hypothesis is that the lipophilic region that is associated with the three‐iodo benzene ring, although shielded by the hydrophilic crown, may show some affinity toward the lipophilic area represented by the tetra‐aza‐cycle in macrocyclic GBCAs.

Neutral Gd‐HPDO3A (chemical structure shown in Figure 1B) was used as the reference GBCA, and the occurrence of binding interactions with the three‐iodo‐benzene‐containing agents was assessed by measuring the proton relaxation enhancement^21^ at 0.5 T on the assumption that the reversible formation of a supramolecular adduct would yield an increase in the molecular reorientation time of the Gd‐water‐proton vector, with a consequent increase in the observed water‐proton relaxation rate.

As shown in Figure 1C, all of the investigated iodinated contrast media (iopamidol, iodixanol, ioversol, iohexol, iobiditrol, and iomeprol at a concentration of 100 mM) yielded an increase in the relaxivity of Gd‐HPDO3A (1 mM). The dimeric iodixanol yielded the highest enhancement.

**FIGURE 2:**
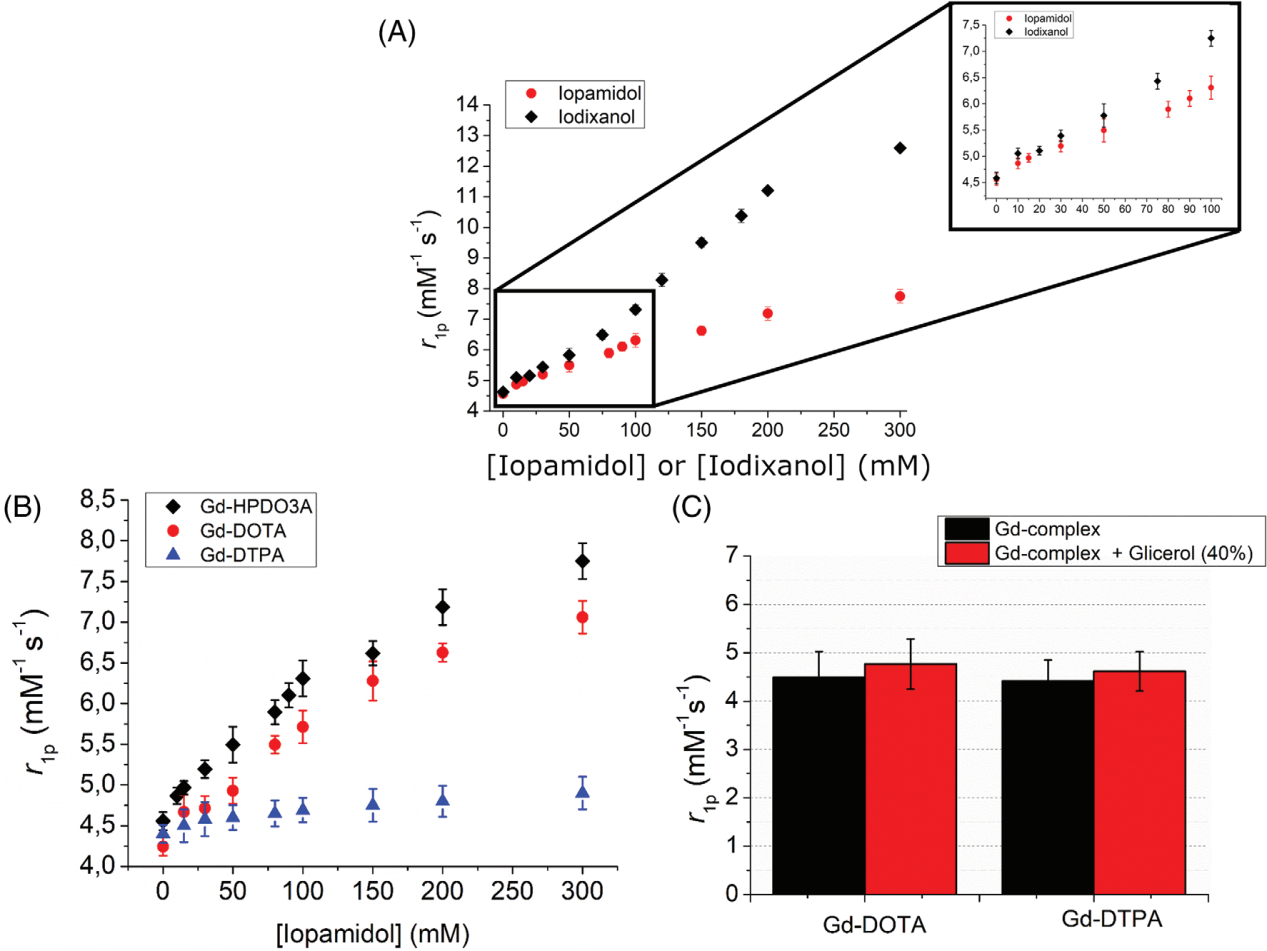
(A) Relaxivity enhancement of Gd‐HPDO3A (1 mM) in the presence of iopamidol or iodixanol at variable concentrations (B_0_ = 0.5 T, pH 7.1 ± 0.1, T = 25°C). (B) Relaxivity enhancement of Gd‐HPDO3A, Gd‐DOTA, and Gd‐DTPA (1 mM) in the presence of iopamidol at variable concentrations (B_0_ = 0.5 T, pH 7.1 ± 0.1, T = 25°C). (C) Relaxivity of Gd‐HPDO3A and Gd‐DOTA (1 mM) in the presence of glycerol 40% (B_0_ = 0.5 T, pH = 7.1 ± 0.1, T = 25°C)

On the basis of these findings, Gd‐HPDO3A was titrated with increasing concentrations of either iopamidol or iodixanol. As reported in Figure 2A, the relaxivity of Gd‐HPDO3A increased from 4.6 to 6.2 and 7.2 mM^−1^ s^−1^ in the presence of excesses of iopamidol and iodixanol (1:100 mol:mol), respectively (measurements carried out at *T* = 25°C).

The measurement of relaxivity in the presence of iopamidol or iodixanol was also carried out at physiological temperature (310 K). As expected for Gd complexes with relatively fast water exchange, the increase of the temperature from 298 K to 310 K resulted in a reduction of the observed relaxivity.

The titration carried out at 310 K (Supporting Information Figure S2) parallels the relavixity enhancement observed at 298 K. In fact, at 310 K, the relaxivity of Gd‐HPDO3A increased from 3.6 to 5.0 and 5.8 mM^−1^ s^−1^ in the presence of a large excesses of iopamidol and iodixanol (1:100 mol:mol), respectively (Supporting Information Figure S2). At both temperatures, approximately 36% and 57% relaxivity enhancement is observed when Gd‐HPDO3A is placed in the presence of a large excess of iopamidol or iodixanol, respectively.

To obtain more insight into the observed behavior, three GBCAs (two macrocyclic and one linear), namely, Gd‐HPDO3A, Gd‐DOTA and Gd‐DTPA, were tested following the addition of iopamidol (chemical structure shown in Figure 1A). There was only a marked increase in relaxivity (Figure 2B) for macrocyclic GBCAs, whereas the linear Gd‐DTPA complex showed a very limited increase.

As one may argue that the increase in microviscosity that was brought about by the highly concentrated iodinated X‐ray CAs may influence the increase in relaxivity, and that this effect could be different for linear and macrocyclic complexes,^8^ the relaxivity was also measured in solutions of Gd‐HPDO3A and Gd‐DTPA, which contained glycerol 40% (vol/vol) to mimic increased viscosity. As reported in Figure 2C, the effect of viscosity appears to be marginal and not significant (∼5% relaxivity increase for both Gd‐HPDO3A and Gd‐DTPA).

**FIGURE 3:**
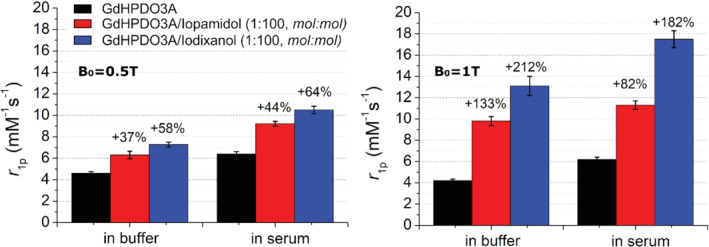
Relaxivity of Gd‐HPDO3A (1 mM) in the presence of iopamidol and iodixanol (100 mM) in either buffer or serum at B_0_ = 0.5 T (A) or B_0_ = 1 T (B) (pH = 7.1 ± 0.1, *T* = 25°C)

Moreover, the effect of the presence of iopamidol and iodixanol was compared in buffer and in human blood serum, at 0.5 T and 1 T (Figure 3A,B, respectively). At 0.5 T, the relaxivity enhancement attained in serum is only slightly higher than that observed in the buffer.

When the relaxivity was measured at 1 T, the formation of the supramolecular adduct in the buffer led to a higher enhancement than was observed at 0.5 T. This enhancement is particularly evident for the adduct formed with iodixanol (*r*_1p_ = 13.2 mM^−1^ s^−1^), compared with iopamidol (*r*_1p_ = 9.8 mM^−1^ s^−1^). In serum, the relaxivity of the adducts is even higher, with the relaxivity values of the specimens with iopamidol and iodixanol being 11.2 and 17.6 mM^−1^ s^−1^, respectively.

### 3.1 In vivo MRI results

The in vitro results were validated in vivo in healthy mice by observing the contrast changes in the kidney regions. These preliminary investigations were limited to an assessment of the effect of iodixanol: the system that yielded the highest response in the in vitro study.

**FIGURE 4:**
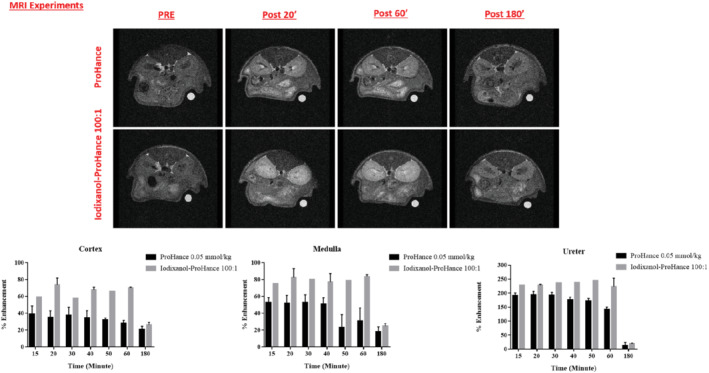
In vivo MR images of the kidney area of healthy mice injected with either Gd‐HPDO3A or the (Gd‐HPDO3A + iodixanol) mixture. Histograms of the superior–inferior enhancements for selected kidneys regions are reported

Magnetic resonance images were acquired on anesthetized mice on an Icon MR scanner (Icon, Bruker) working at 1 T. The MRI acquisitions were performed *before* and 20, 60, and 180 min *after* injection, to visualize the enhancement caused by the co‐presence of iodixanol and GdHPDO3A compared with Gd‐HPDO3A alone at the dose of 0.05 mmol/kg. Figure 4 summarizes the obtained results and reports the percentage of enhanced signal observed in the different kidney regions (cortex, medulla, and ureters/renal pelvis; an example of ROIs is reported in Supporting Information Figure S1).

The in vivo results are fully consistent with the in vitro observations, as the co‐presence of Gd‐HPDO3A and iodixanol resulted in a marked increase in the signal intensities recorded in the three kidney regions. Overall, the longer persistence of the enhancement in the case of the co‐presence of the two agents, compared with the incipient washout of Gd‐HPDO3A, is consistent with the slower excretion of iodixanol.

### 3.2 In vivo CT results

**FIGURE 5:**
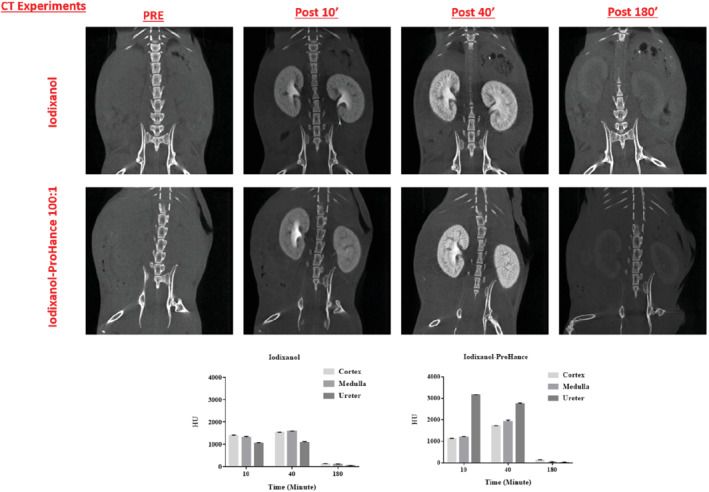
Serial in vivo CT images (75 Kv, with 512 views and ×2.3 magnification) of the kidney area of healthy mice injected with either iodixanol or with the (Gd‐HPDO3A + iodixanol) mixture. Histograms of the density contrast (in Hounsfield units) for selected kidney regions are reported

As for MRI acquisition, the 2D images (sagittal view) of the kidneys from the anesthetized mice were acquired on a GMI‐Triumph trimodality preclinical scanner, *before* and 10, 40, and 180 min *after* injection of either 4.0 g/kg iodixanol or iodixanol/Gd‐HPDO3A mixture (4.0 g/kg/0.05 mmol/kg) (Figure 5).

The co‐presence of Gd‐HPDO3A and iodixanol also provided the best contrast for distinguishing the anatomical structure of the kidneys (cortex, medulla, and ureters/renal pelvis; an example of ROIs is reported in Supporting Information Figure S1) in the CT images, and, in particular, generated a difference in the densities of the ureters/renal pelvis and the less‐dense cortex and medulla (increased density of about 3000 HU 10 min after injection into the ureters/renal pelvis).

## 4 DISCUSSION

The results reported herein show the detection of relaxation enhancements of about 30% and 40% for 1‐mM solutions of Gd‐HPDO3A in the presence of a large excess of iopamidol and iodixanol, respectively. The absence of such a relaxation enhancement in the case of the linear Gd‐DTPA complex strongly supports the view that the observed effect is related to the reversible formation of supramolecular adducts between the macrocyclic GBCAs and the three‐iodo‐containing aromatic moieties of the X‐ray CAs. Simple viscosity effects that would have affected the molecular reorientation time of GBCAs^8^ have been ruled out by the relaxometric measurements carried out in highly viscous glycerol‐containing solutions. For this purpose, a 40% glycerol solution in water was used, as it has viscosities of approximately 3.7 cP at 20°C and 2.3 cP at 37°C.^22^ The viscosity obtained under these conditions is even higher than the one displayed by the maximum concentration of iopamidol used in this work (i.e., 300 mM of iopamidol, corresponding to 115 mgI/ml).^23^, ^24^ A similar viscosity is associated to a much more concentrated solution of iopamidol (e.g., iopamidol 41% [i.e., 524 mM of iopamidol, corresponding to 200 mgI/ml], which has viscosities of 3.3 cP and 2 cP at 20°C and 37°C, respectively).

The detection of a relaxation enhancement following the co‐presence of iodinated and macrocyclic GBCAs may be a useful way to obtain more insight into their in vivo biodistribution and excretion pathways, and for delineating detailed structural features in the excretory organs. In this context, one may envisage studies that aim to assess how changes in the biodistribution of the two types of agents may be affected by pathological states. The high resolution associated with detecting changes in their relative amounts using MRI may reveal new factors in the considered diseases that could not be evidenced using a single agent.

More generally, these findings call for attention to be paid to possible hydrophobic interactions that occur between the tetra‐aza cycle in macrocyclic GBCAs and endogenous and exogenous species.

## Supporting information


**Figure S1.** Chart representing a CT image of cortex (red region of interest [ROI]), medulla (blue ROI), and ureters/renal pelvis (yellow ROI)
**Figure S2**. Relaxivity enhancement of Gd‐HPDO3A (1 mM) in the presence of iopamidol or iodixanol at variable concentrations (B_0_ = 0.5 *T*, pH 7.1 ± 0.1) at *T* = 25°C and *T* = 37°C
