# Supplementary material for: The interaction between iodinated X‐ray contrast agents and macrocyclic GBCAs provides a signal enhancement in T1‐weighted MR images: Insights into the renal excretion pathways of Gd‐HPDO3A and iodixanol in healthy mice
Source: Magn Reson Med. 2022 Mar 7;88(1):357–64. doi: 10.1002/mrm.29190 (PMC9314041; doi:10.1002/mrm.29190)
Supplement: Supplementary file 1 — Figure S1. Chart representing a CT image of cortex (red region of interest [ROI]), medulla (blue ROI), and ureters/renal pelvis (yellow ROI) Figure S2. Relaxivity enhancement of Gd‐HPDO3A (1 mM) in the presence of iopamidol or iodixanol at variable concentrations (B0 = 0.5 T, pH 7.1 ± 0.1) at T = 25°C and T = 37°C [file MRM-88-357-s001.docx]

**Supporting information for**

**The interaction between iodinated X-ray Contrast Agents and macrocyclic GBCAs provides a signal enhancement in *T*_1w_-MR images. Insights into the renal excretion pathways of Gd-HPDO3A and iodixanol in healthy mice.**

*Enza Di Gregorio ^1,§^ , Francesca Arena ^1,§^ , Eliana Gianolio ^1^ , Giuseppe Ferrauto ^1,*^, Silvio Aime ^2^ .*

*^1^ Molecular Imaging Center, Dept. of Molecular Biotechnology and Health Sciences, University of Turin (It)*

*^2^ IRCCS SDN, Via E. Gianturco 113, 80143 Napoli, Italy*

*^§^ These authors equally contributed*

*^*^ Corresponding author: Dr. Giuseppe FERRAUTO, Molecular Imaging Center, Dept. of Molecular Biotechnology and Health Sciences, University of Turin (It). giuseppe.ferrauto@unito.it +390116708459*

**Supplementary images**

**
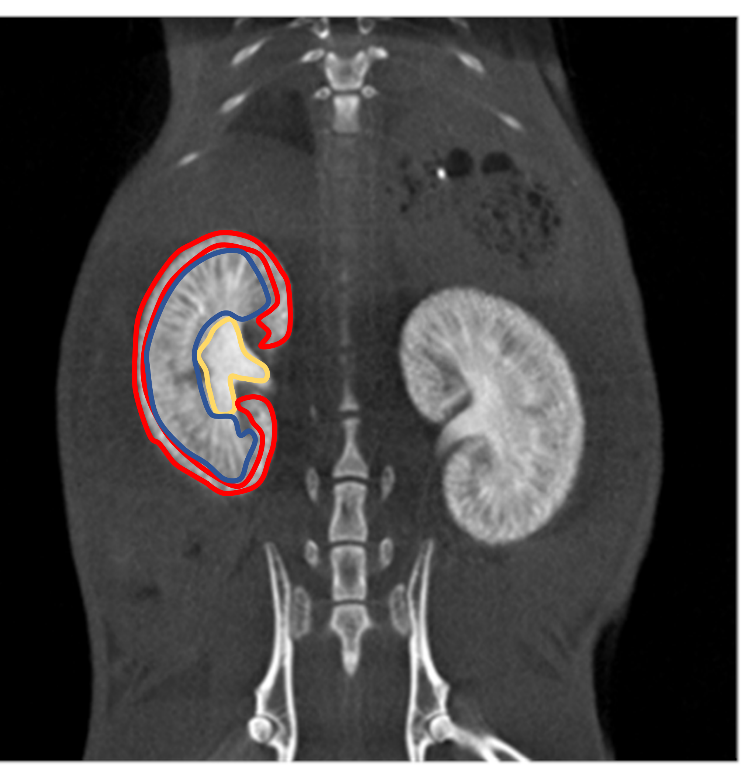
**

**Fig.S1** Chart representing a CT image of cortex (*red ROI*), medulla (*blue ROI*), and ureters/renal pelvis (*yellow ROI*).

**

**

**Fig.S2** Relaxivity enhancement of Gd-HPDO3A (1mM) in the presence of iopamidol or iodixanol at variable concentrations (B_0_=0.5 T, pH 7.1±0.1) at T=25° C and T=37°C.
